# Assessment of Combination of Automated Pupillometry and Heart Rate Variability to Detect Driving Fatigue

**DOI:** 10.3389/fpubh.2022.828428

**Published:** 2022-02-21

**Authors:** Lin Shi, Leilei Zheng, Danni Jin, Zheng Lin, Qiaoling Zhang, Mao Zhang

**Affiliations:** ^1^Department of Emergency Medicine, Second Affiliated Hospital, Zhejiang University School of Medicine, Hangzhou, China; ^2^Key Laboratory of the Diagnosis and Treatment of Severe Trauma and Burn of Zhejiang Province, Hangzhou, China; ^3^Zhejiang Province Clinical Research Center for Emergency and Critical Care Medicine, Hangzhou, China; ^4^Department of Psychiatry, Second Affiliated Hospital, Zhejiang University School of Medicine, Hangzhou, China

**Keywords:** driving fatigue, traffic safety, pupillary light reflex (PLR), heart rate variability, automated pupillometry

## Abstract

**Objectives:**

Approximately 20~30% of all traffic accidents are caused by fatigue driving. However, limited practicability remains a barrier for the real application of available techniques to detect driving fatigue. Use of pupillary light reflex (PLR) may be potentially effective for driving fatigue detection.

**Methods:**

A 90 min monotonous simulated driving task was utilized to induce driving fatigue. During the task, PLR measurements were performed at baseline and at an interval of 30 min. Subjective rating scales, heart rate variability (HRV) were monitored simultaneously.

**Results:**

Thirty-two healthy volunteers in China participated in our study. Based on the results of subjective evaluation and behavioral performances, driving fatigue was verified to be successfully induced by a simulated driving task. Significant variations of PLR and HRV parameters were observed, which also showed significant relevance with the change in Karolinska Sleepiness Scale at several timepoints (|*r*| = 0.55 ~ 0.72, *P* < 0.001). Furthermore, PLR variations had excellent ability to detect driving fatigue with high sensitivity and specificity, of which maximum constriction velocity variations achieved a sensitivity of 85.00% and specificity of 72.34% for driving fatigue detection, vs. 82.50 and 78.72% with a combination of HRV variations, a nonsignificant difference (AUC = 0.835, 0.872, *P* > 0.05).

**Conclusions:**

Pupillary light reflex variation may be a potential indicator in the detection of driving fatigue, achieving a comparative performance compared with the combination with heart rate variability. Further work may be involved in developing a commercialized driving fatigue detection system based on pupillary parameters.

## Introduction

Road traffic accidents has become the eighth leading cause of death worldwide (1). They killed nearly 1.35 million people every year, and caused up to 50 million injuries (1), resulting in huge losses for societies with regard to population health and economic matters. However, ~20~30% of all traffic accidents are caused by fatigue driving, which is in fact preventable (2). Driving fatigue is often induced by prolonged driving without due rest, being presented with deteriorated concentration, low alertness, slow reaction to emergencies, etc., all of which can lead to road traffic crashes (3). Continuous efforts have been made to develop reliable indicators of driving fatigue to warn drivers of their fatigue status promptly and thus preventing possible accidents from occurring (4–10).

Among these exploratory studies, the detection systems incorporating physiological signals of drivers, which mainly involves extracting and analyzing characteristics of electroencephalogram (EEG) (6, 11–13), heart rate variability (HRV) (5, 14, 15), electromyogram (EMG) (7, 16), etc., are considered as the most accurate and reliable ones to reflect the mental states. However, due to the inconvenience to wear, and various complicated algorithms, the real application of these techniques still remains a challenge. In contrast, eye metrics also offers a promising method for monitoring fatigue (17), including the percentage of eyelid closure (8, 18), blink-based (19) and pupil-based features (10, 20, 21) in an eyewear system. These eye-tracking data provide additional evidence for motivational disengagement, describing the effect of fatigue on attention and task performance (22). However, detection systems using a camera-based approach has a limitation of illumination, and most of those need computers, image processing algorithms and feature extraction techniques to extract drowsy symptoms (19). The effectiveness of pupillary light reflex (PLR) on the assessment of sleep deprivation was recently explored (23), suggesting that PLR variables might also be able to indicate fatigue. Automated pupillometry, which has been utilized in different clinical settings (24, 25), enables to provide several accurate and reliable parameters with respect to pupil size and PLR (26, 27). To date, no study has been conducted to analyze the association between PLR variations with driving fatigue.

Our aim was to investigate the effectiveness of quantitative PLR on driving fatigue detection, as well as the possible detection performances of a combination of PLR and HRV, which may very likely promote the further development of a driving fatigue detection system in the future.

## Materials and Methods

### Study Population

We conducted a single center, prospective, observational diagnostic study using a volunteer sample between November, 2020, to April, 2021. Thirty-two healthy postgraduate students from Zhejiang University School of Medicine in China voluntarily participated in our study, among whom 20 were male. All participants held valid driving license of more than 2 years, with at least half of a year driving experience, had regular sleep pattern, normal or corrected to normal vision and no history of any psychiatric disorder. All participants were asked to follow the below requirements before the tests: (1) refrain from alcohol, caffeine and tea within 12 h; (2) adequate sleep (almost 6~8 h) the day before the experiment; (3) wash the hair within 24 h. The study was approved by the ethics committee of Second Affiliated Hospital of Zhejiang University School of Medicine (approval number: 2020-893). Informed written consent and training were provided prior to entering the experiment.

### Driving Fatigue Induction

Among all recruited participants, a 90 min monotonous simulated driving task was conducted in a darkened room, involving in a simple driving simulator (Nanjing Shengguan Jinbang Electronic Technology, China). A straight and monotonous route with low traffic density was designed in advance. The performance method in the simulator was the same as that in a real car. The visual display of the driving simulated environment was a 14-inch liquid crystal display at 80 cm in the front of the subjects' eyes. Current speed and car gear were showed on the screen, and the engine noises as well as nearby traffic noises were provided. During the driving task, the subjects were asked to restrict all unnecessary movements and drive in the center of the road, maintaining a constant speed between 70 and 90 km/h.

**Figure 1:**
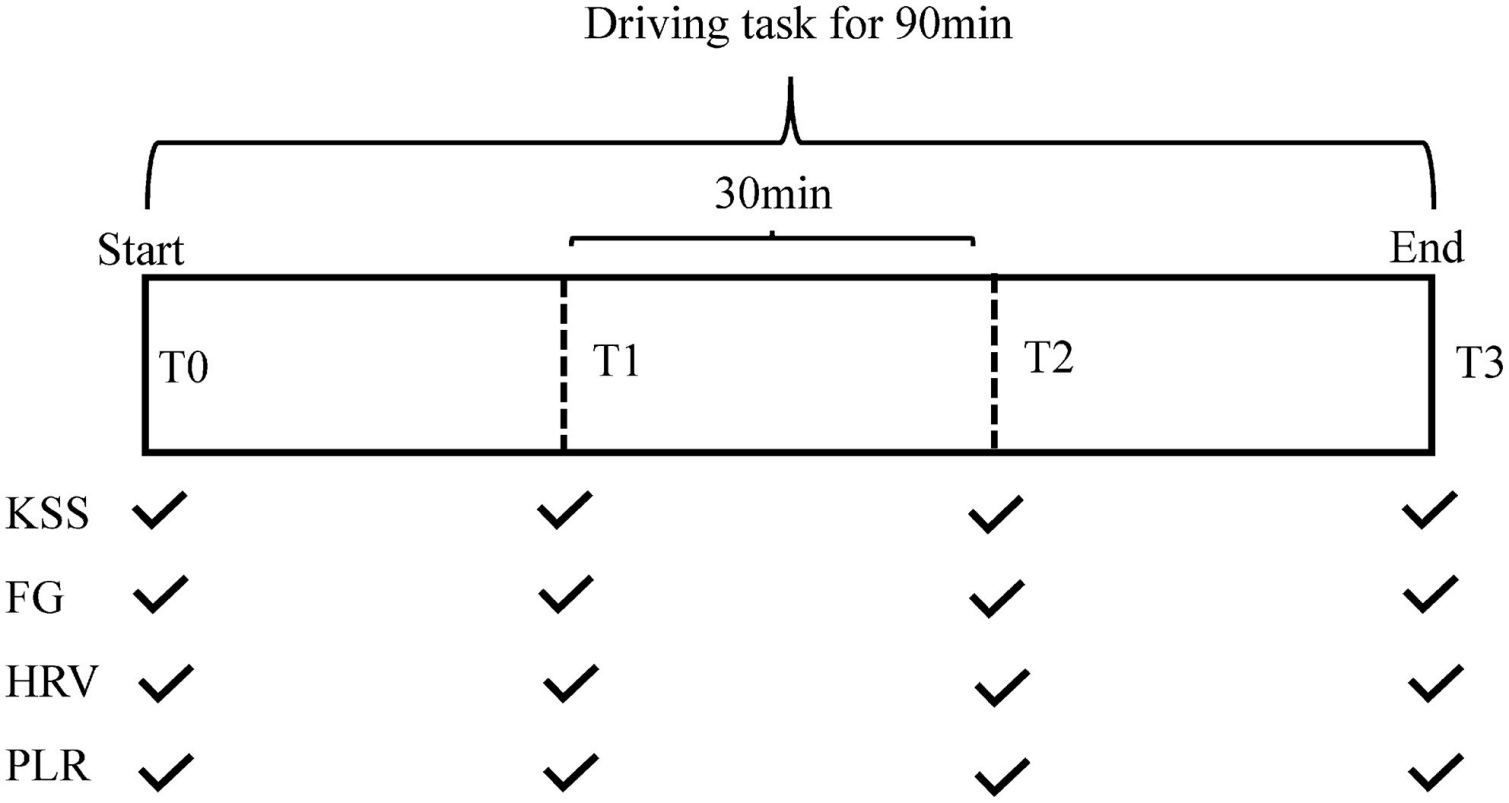
Schematic diagram for the entire experimental framework. FG, fatigue grade; HRV, heart rate variability; KSS, Karolinska Sleepiness Scale; PLR, pupillary light reflex.

The experiment started between 9:00 and 11:00 a. m. or 3:00 and 5:00 p. m. for every subject to minimize the effect of circadian variance. After 10 min of rest, 3 min of HRV were recorded, which was regarded as the baseline data (T0). Meanwhile, subjective assessments and pupillary measurements were taken. In order to evaluate fatigue level over the course of the experiment, the task was divided into three 30-min sections. When each section was completed, the HRV and PLR data were recorded. Meanwhile, subjective ratings were performed. The flowchart of the study was shown in Figure 1.

### Subjective Assessments

Subjects completed the Karolinska Sleepiness Scale (KSS) (28), which is a nine-point scale for assessing sleepiness with responses ranging from extremely alert (1) to very sleepy (9). Driving fatigue was defined as KSS ≥ 7. Fatigue Grade (FG) scale, which is a five-point scale designed by our team with responses ranging from no fatigue (0) to severe fatigue (5), was also completed.

### Heart Rate Variability Data Acquisition

HRV was assessed with a protocol of 3 min sampling using the SA-3000P (Medi-core, Korea). Subjects were instructed to stay with eyes open, be silent, and breath normally during measurement. Standard deviation of the NN intervals (SDNN) and frequency domain indicators including low frequency (LF, 0.04~0.15 Hz), high frequency (HF, 0.15~0.4 Hz), normalized LF (LFnu), normalized HF (HFnu), LF/HF ratio were measured and showed automatically on the screen. The SDNN, LFnu, HFnu, and LF/HF were collected for further analysis.

### Pupillary Light Reflex Data Acquisition

PLR were measured using the PLR-3000 pupillometer (NeurOptics, CA, USA). It was performed with a rubber cup covering the measured eye and the subject's hand covering the non-measured eye. A flash of visible white light with a duration of 0.8 s and a pulse intensity of 120 μW was delivered to induce a pupillary reflex, and repeated video images at more than 30 frames/s were stored for 3.21 s. The device provided with maximum and minimum pupil size (Max and Min), constriction percentage (%PLR), latency (LAT), constriction and dilation velocity (CV and DV), and T75. PLR measurement was performed on each eye for a series of three stimuli, then the data was averaged, producing one PLR data point for each subject. Maintaining eye opening during the recording was encouraged, but if interrupted, the test would be repeated after 5 s rest.

### Statistical Analysis

Normal distribution was confirmed with the Kolmogorov–Smirnov test. For normally or non-normally distributed continuous variables, results are given as mean ± SD or median (IQR), respectively. Repeated measures analysis of variance or Friedman test was used to test the variability of different approaches. When the main effect was significant, a *post-hoc* analysis with Bonferroni correction was conducted. Correlations were determined by Spearman analysis. Considering the between-subject differences at baseline, differences between the baseline and the measurement at each time point were utilized to conduct the Spearman's correlation. Due to there are a total of 24 correlational analyses and the *P*-values have to be adjusted for the number of tests, *P* < 0.002 (0.05/24) of two-sided was considered to be significant. Detective performances of each indicator and the combination of two indicators were analyzed by calculating specificity, sensitivity, and the area under the receiver operating characteristic curve (AUC). *P* < 0.05 of two-sided was considered to be significant. All analyses were performed using Prism software (version 7.0; GraphPad, USA) and Medcalc software (version 19.0; Belgium).

## Results

### Subjective Rating Results and Behavioral Performances

The subjects' mean ± SD age was 25.5 ± 3.1 years, sleeping hours in the past 24 h were 7.4 ± 0.9 h, driving experience was 4.3 ± 1.9 years. To test whether our fatigue model was successful, we analyzed the subjective fatigue ratings and behavioral performances. Compared to the beginning of the experiment, we found significantly higher fatigue scores after the experiment [KSS: T0: 3 (2–4), T1: 5 (4–6) ^a^, T2: 6 (5.25–7) ^ab^ vs. T3: 7 (6–8) ^ab^, *P* < 0.001; FG scale: T0: 0, T1: 0 (0–1) ^a^, T2: 2 (1.25–3) ^a^ vs. T3: 3 (2.25–4) ^abc^, *P* < 0.001]. Additionally, the number of accidents increased significantly from the beginning to the end of the driving task [T0: 0, T1: 1 (0–1), T2: 1 ^a^ vs. T3: 2 (2, 3) ^abc^, *P* < 0.001], indicating that the manipulation was successful (note: ^a^*P* < 0.05 vs. T0; ^b^*P* < 0.05 vs. T1; ^c^*P* < 0.05 vs. T2).

### Fluctuations of Heart Rate Variability

**Figure 2:**
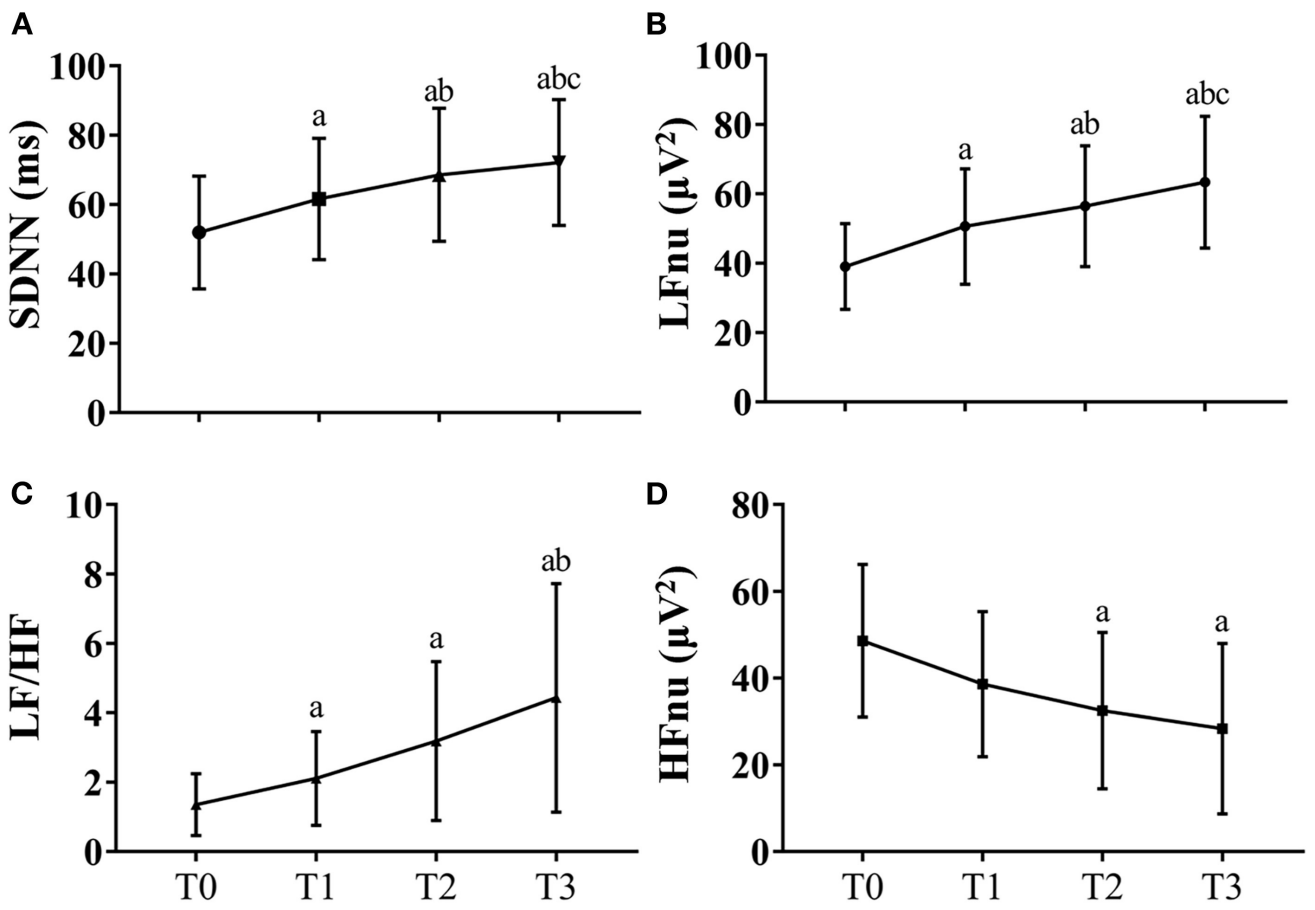
HRV fluctuations during the driving task for all participants. Due to missing data of HRV of two participants, the data at these two timepoints were the results of data analysis of 30 participants. The error bars in this figure represent mean ± SD. **(A)** The variations of SDNN for HRV. **(B)** The variations of LFnu for HRV results. **(C)** The variations of LF/HF for HRV results. **(D)** The variations of HFnu for HRV. HF indicates high frequency; HFnu, normalization of HF power by the formula: HF / (LF + HF) * 100; HRV, heart rate variability; KSS, Karolinska Sleepiness Scale; LF, low frequency; LFnu, normalization of LF power by the formula: LF / (LF + HF) * 100; SDNN, standard deviation of the NN intervals. ^a^*P* < 0.05 vs. T0; ^b^*P* < 0.05 vs. T1; ^c^*P* < 0.05 vs. T2.

Significant differences in HRV indicators between the first and last section of driving task were observed. Mean SDNN increased across long-term driving (T0: 39.04 ± 12.33 ms, T1: 50.60 ± 16.63 ms ^a^, T2: 56.43 ± 17.41 ms ^ab^ vs. T3: 63.34 ± 19.00 ms ^abc^, *P* < 0.001, Figure 2A). The mean power of HRV in LFnu and LF/HF also significantly increased in a linear fashion [LFnu: T0: 51.96 ± 16.25 μV^2^, T1: 61.63 ± 17.51 μV^2a^, T2: 68.58 ± 19.24 μV^2ab^ vs. T3: 72.12 ± 18.16 μV^2abc^; LF/HF: T0: 1.23 (0.68–1.70), T1: 1.91 (0.97–3.19) ^a^, T2: 2.63 (1.06–4.66) ^a^ vs. T3: 3.29 (1.49–7.57) ^ab^; both *P* < 0.001, Figures 2B,C], while there was a mild decline in the mean power of HRV in HFnu, from T0: 44.99 (37.06–59.92) μV^2^, T1: 35.68 (23.81–51.24) μV^2^, T2: 29.05 (16.24–49.71) μV^2a^ to T3: 22.94 (11.46–44.15) μV^2a^ (*P* < 0.001, see Figure 2D) (note: ^a^*P* < 0.05 vs. T0; ^b^*P* < 0.05 vs. T1; ^c^*P* < 0.05 vs. T2).

### Variations of Quantitative Pupillary Light Reflex

With the increase of driving hours, Min significantly decreased and %PLR increased (Min: T0: 4.40 ± 0.7 mm, T1: 4.20 ± 0.7 mm ^a^, T2: 4.08 ± 0.7 ^ab^ vs. T3: 3.99 ± 0.6 mm ^ab^; %PLR: T0: 36.1 ± 4.6 %, T1: 37.4 ± 4.2 % ^a^, T2: 38.9 ± 4.6 % ^ab^ vs. T3: 39.7 ± 3.8 % ^ab^; both *P* < 0.001, Table 1). Similarly, significant increases in ACV and MCV were also observed with the increase of the driving hours (ACV: T0: 3.20 ± 0.43 mm/s, T1: 3.34 ± 0.46 mm/s ^a^, T2: 3.50 ± 0.44 mm/s ^ab^ vs. T3: 3.68 ± 0.47 mm/s ^abc^; MCV: T0: 5.19 ± 0.74 mm/s, T1: 5.53 ± 0.75 mm/s ^a^, T2: 5.72 ± 0.74 mm/s ^ab^ vs. T3: 5.90 ± 0.71 mm/s ^ab^; both *P* < 0.001, Table 1). However, the differences in Max, Lat, ADV and T75 were not statistically significant at different timepoints (note: ^a^*P* < 0.05 vs. T0; ^b^*P* < 0.05 vs. T1; ^c^*P* < 0.05 vs. T2).

**Table 1:** The results of PLR for both eyes through the whole process of driving task.

**Variables**	**T0 (*n* = 31)**	**T1 (*n* = 31)**	**T2 (*n* = 31)**	**T3 (*n* = 31)**	***P-*value**
Max, mean ± SD, mm	6.88 ± 0.7	6.74 ± 0.7^a^	6.70 ± 0.7^a^	6.76 ± 0.7^a^	<0.001
Min, mean ± SD, mm	4.40 ± 0.7	4.20 ± 0.7^a^	4.08 ± 0.7^a^^b^	3.99 ± 0.6^a^^b^	<0.001
%PLR, mean ± SD, %	36.1 ± 4.6	37.4 ± 4.2^a^	38.9 ± 4.6^a^^b^	39.7 ± 3.8^a^^b^	<0.001
Lat, median (IQR), s	0.23 (0.21 ~ 0.25)	0.23 (0.21 ~ 0.25)	0.22 (0.21 ~ 0.25)	0.22(0.21 ~ 0.23)	0.11
ACV, mean ± SD, mm/s	3.20 ± 0.43	3.34 ± 0.46^a^	3.50 ± 0.44^a^^b^	3.68 ± 0.47^a^^b^^c^	<0.001
MCV, mean ± SD, mm/s	5.19 ± 0.74	5.53 ± 0.75^a^	5.72 ± 0.74^a^^b^	5.90 ± 0.71^a^^b^	<0.001
ADV, mean ± SD, mm/s	1.37 ± 0.16	1.40 ± 0.20	1.42 ± 0.22	1.42 ± 0.22	0.812
T75, median (IQR), s	2.69 (2.15 ~ 3.14)	2.54 (2.08 ~ 3.25)	2.73 (2.35 ~ 3.37)	2.70 (2.40 ~ 3.14)	0.084

*ACV, average constriction velocity; ADV, average dilation velocity; Lat, latency; MCV, maximum constriction velocity; Min, minimum pupil size; %PLR, constriction percentage; T75, time to 75% recovery*.

*Due to missing data of PLR of one participant, the data at all timepoints were the results of data analysis of 31 participants. Values are presented as mean ± SD for normally distributed data or as median (IQR) for non-normally distributed data*,

a *P < 0.05 vs. T0*;

b *P < 0.05 vs. T1*;

c *P < 0.05 vs. T2*.

### Correlation Analysis Between Different Methods

**Figure 3:**
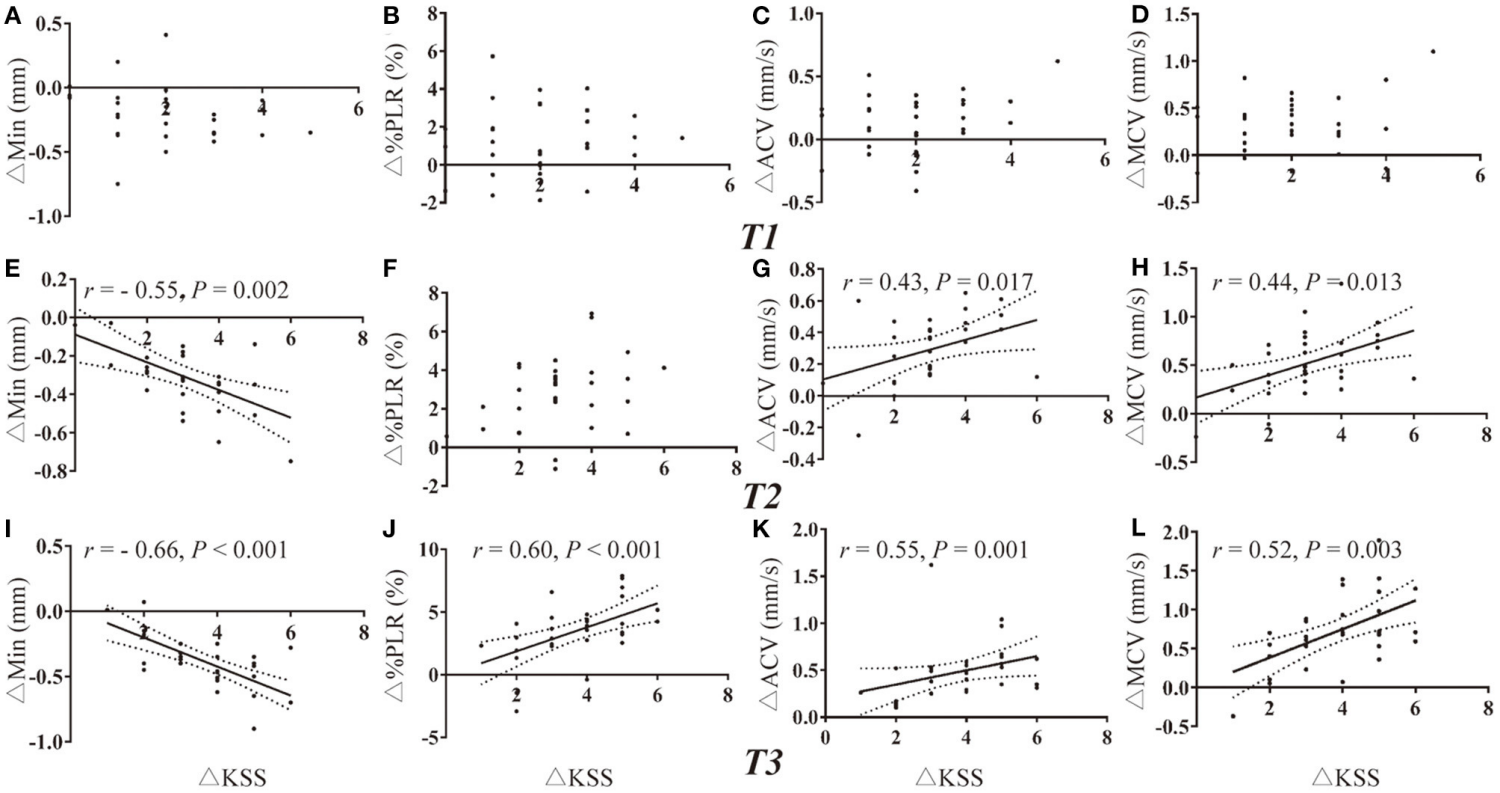
Correlations of PLR variations with the change in KSS. **(A–D)** Correlation of ΔKSS with the change in PLR parameters at T1. **(E–H)** Correlation of ΔKSS with the change in PLR parameters at T2. **(I–L)** Correlation of ΔKSS with the change in PLR parameters at T3. ACV, average constriction velocity; KSS, Karolinska Sleepiness Scale; MCV, maximum constriction velocity; Min, minimum pupil size; %PLR, constriction percentage; Δ, differences in all variables from baseline to the measurement at each timepoint.

**Figure 4:**
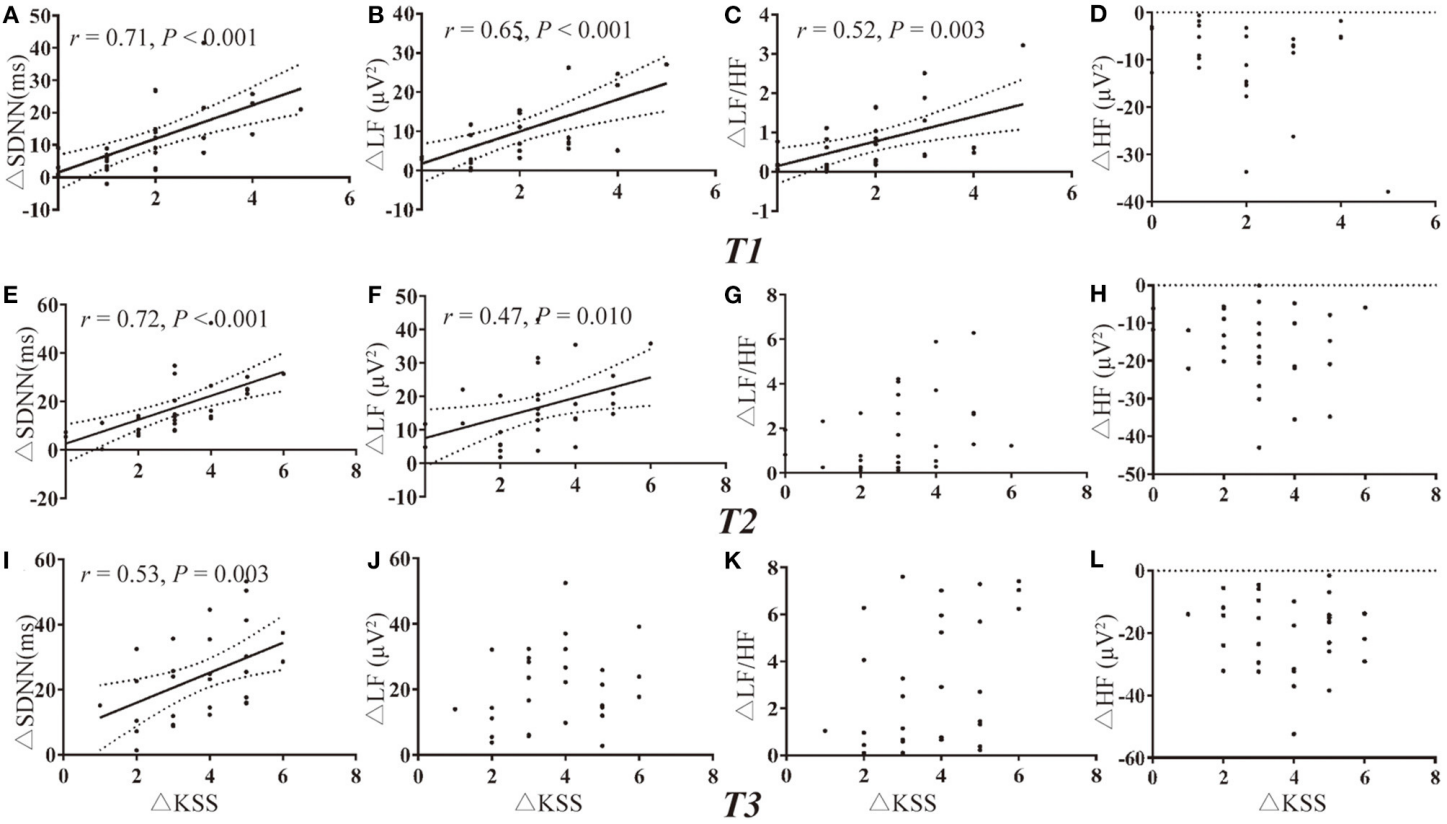
Correlations of HRV variations with the change in KSS. **(A–D)** Correlation of ΔKSS with the change in HRV parameters at T1. **(E–H)** Correlation of ΔKSS with the change in HRV parameters at T2. **(I–L)** Correlation of ΔKSS with the change in HRV parameters at T3. HF indicates high frequency; HFnu, normalization of HF power by the formula: HF / (LF + HF) * 100; HRV, heart rate variability; KSS, Karolinska Sleepiness Scale; LF, low frequency; LFnu, normalization of LF power by the formula: LF / (LF + HF) * 100; SDNN, standard deviation of the NN intervals; Δ, differences in all variables from baseline to the measurement at each timepoint.

Due to the missing data of PLR of one participant and missing data of HRV of two participants, there were a total of 31 and 30 values for deprived PLR and HRV parameters for correlation analysis at each timepoint. Change in KSS (ΔKSS) were not correlated with the change in PLR parameters at T1 and T2 (all *P* > 0.002, Figures 3A–H). Change in KSS (ΔKSS) was moderately correlated with the change in Min (ΔMin), %PLR (Δ%PLR) and ACV(ΔACV) (*r* = −0.66, 0.60, 0.55, all *P* < 0.001, Figures 3I–K) at T3. Similarly, ΔKSS was highly correlated with the change in SDNN (ΔSDNN) at T1 and T2 (*r* = 0.71 and 0.72, both *P* < 0.001, Figures 4A,E), and moderately correlated with change in LFnu (ΔLF) at T1 (*r* = 0.65, *P* < 0.001, Figure 4B). However, ΔKSS was not correlated with change in LF/HF (ΔLF/HF) and HF (ΔHF) at T1 (Figures 4C,D), ΔLF, ΔLF/HF and ΔHF at T2 (Figures 4F–H), change in all HRV parameters at T3 (Figures 4I–L)

### Performance of Pupil Light Reflex and Heart Rate Variability Variations in Driver Fatigue Detection

**Figure 5:**
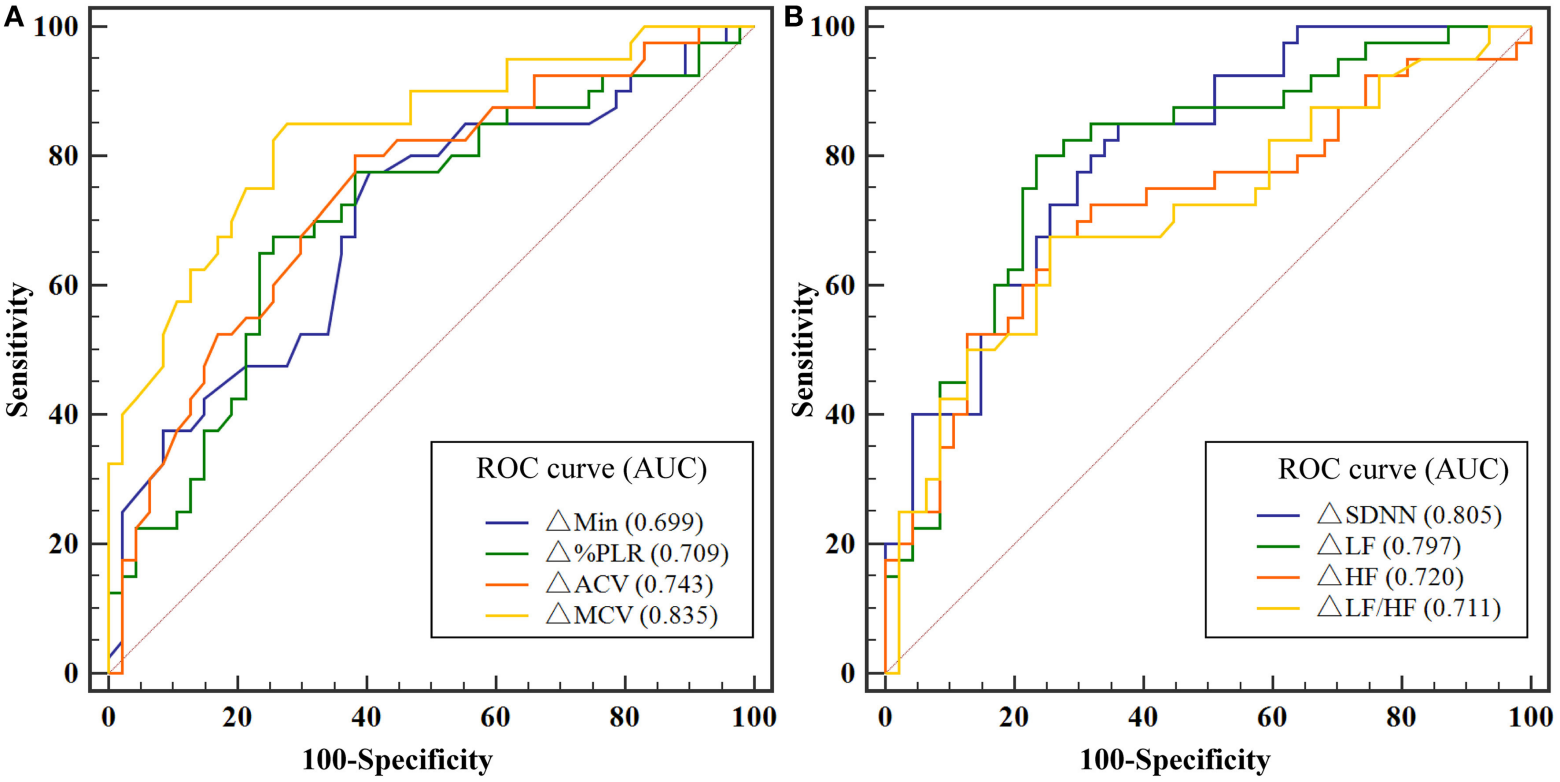
**(A)** ROC analysis for the detection of driving fatigue with PLR variations. **(B)** ROC analysis for the detection of driving fatigue with HRV variations. Due to missing data of HRV of two participants, and missing data of PLR of one participant, the data of 29 participants were used for ROC analysis. ACV, average constriction velocity; AUC, area under the curve; Min, minimum pupil size; HF indicates high frequency; HFnu, normalization of HF power by the formula: HF / (LF + HF) * 100; HRV, heart rate variability; KSS, Karolinska Sleepiness Scale; LF, low frequency; LFnu, normalization of LF power by the formula: LF / (LF + HF) * 100; MCV, maximum constriction velocity; ROC, receiver operating characteristic curve; SDNN, standard deviation of the NN intervals; %PLR, constriction percentage; Δ, differences in all variables from baseline to the measurement at each timepoint.

ROC analysis showed that both PLR and HRV variations had a significant discriminatory power to detect driver fatigue (Table 2), which was defined as KSS more than seven in the present study. The Youden index identified a ΔMCV cut-off of 0.43 mm/s for driving fatigue detection with a sensitivity of 85.00% and a specificity of 72.34% (AUC = 0.835, *P* < 0.001, Figure 5A). Similarly, best cut-off of ΔSDNN was > 13.01 ms, with an AUC of 0.805 (*P* < 0.001, sensitivity 85.00%, specificity 63.83%, Figure 5B). A combination of ΔMCV and ΔSDNN achieved an AUC of 0.872 (*P* < 0.001, sensitivity 82.50%, specificity 78.72%), yet no significant differences were found among the performances of ΔMCV, ΔSDNN and their combination (all *P* > 0.05, Delong test).

**Table 2:** The characteristics of ROC curves.

**Variables**	***AUC***	***95%CI***	***P-*value**	**Cutoff**	**Sensitivity**	**Specificity**
**PLR variations**						
ΔMin	0.699	0.592 ~ 0.793	< 0.001	< −0.29 mm	77.50%	59.57%
Δ%PLR	0.709	0.601 ~ 0.801	< 0.001	> 3.19 %	67.50%	74.47%
ΔACV	0.743	0.638 ~ 0.830	< 0.001	> 0.26 mm/s	80.00%	61.70%
ΔMCV	0.835^a^	0.740 ~ 0.906	< 0.001	> 0.43 mm/s	85.00%	72.34%
**HRV variations**						
ΔSDNN	0.805	0.706 ~ 0.882	< 0.001	> 13.01 ms	85.00%	63.83%
ΔLFnu	0.797	0.697 ~ 0.876	< 0.001	> 14.32 μV^2^	80.00%	76.60%
ΔHFnu	0.720	0.614 ~ 0.811	< 0.001	< −14.59 μV^2^	67.50%	74.47%
ΔLF/HF	0.711	0.604 ~ 0.803	< 0.001	> 1.15	67.50%	74.47%
**Combinations of variables for PLR and HRV**						
ΔMCV + ΔSDNN	0.872	0.784 ~ 0.934	<0.001	/	82.50%	78.72%

ACV, average constriction velocity; AUC, area under curve; HF, high frequency; HFnu, normalization of HF power by the formula: HF / (LF + HF) * 100; HRV, heart rate variability; LF, low frequency; LFnu, normalization of LF power by the formula: LF / (LF + HF) * 100; MCV, maximum constriction velocity; Min, minimum pupil size; SDNN, standard deviation of the NN intervals. %PLR, constriction percentage; Δ, differences in all variables from baseline to the measurement at each timepoint;

a *P < 0.05 vs. ΔMin*.

## Discussion

This study investigated the effectiveness of pupillary light reflex and heart rate variability in driving fatigue detection. The findings are as follows: (1) significant increases in several variables of PLR and HRV were observed at fatigue state; (2) the variations of PLR and HRV showed significant relevance with the change in KSS; (3) PLR variations had excellent ability to detect driving fatigue, with a comparative performance with HRV or a combination of PLR and HRV.

In our study, a simple driving simulator was applied to induce driving fatigue. For safety reasons, experimental tests for the fatigue detection are often conducted on driving simulators in a controlled environment. Previous literature suggested that driving fatigue could occur in a monotonous driving environment with a duration of 90 min (3, 13, 14). Among many endogenous factors, time-on-task and high workload accumulate mental fatigue, while the sleep-related causal factors worsen sleepiness (29). In our study, all participants were asked to refrain from alcohol, caffeine and tea within 12 h and obtain adequate sleep (almost 6~8 h) the day before the experiment to avoid the sleep-related causal factors. Then, based on the increased scores of subjective ratings and deteriorated driving performances of subjects, a conclusion could be drawn that a 90-min driving task successfully induced driving fatigue.

It has been indicated that HRV is the most sensitive index assessing the regulation between sympathetic and parasympathetic nervous systems (15). An increase in SDNN, LFnu, LF/HF ratio, and a decrease in HFnu in this study corroborates earlier findings (14). The results implied that the dominating activity turned from parasympathetic to sympathetic activity. In the first section, without obvious fatigue, sympathetic activity increased due to the task of simulated driving. With fatigue increased and performance deteriorated, participants counteracted the sleep demand by trying to stay awake to complete the task, resulting in consistent sympathetic activation.

Most importantly, significant differences of pupillary parameters between different periods of task were found. For the automated pupillometer, %PLR depends on the intensity and duration of the stimulus (30), while CV are related to reflex amplitude except in cases of unusual pupillary syndromes (30). With identical intensities and durations of all stimuli in our study, the PLR variations were associated with the occurrence of fatigue. Sympathetic and parasympathetic fibers dictate the pupil diameter to contract or dilate by balanced activation of the dilator and sphincter pupillae muscles in changing the light exposures toward the eyes (17). When fatigued, sympathetic activity would increase, with greater contraction in a shorter time to respond to the light stimulus, which might explain the variations of PLR. In addition, the measurements reported were not independent. As the parameters of Max and Lat were unchanged across the four measurements, the increase in the %PLR would result in an increase in velocity.

To validate the effectiveness of HRV and PLR variations on driving fatigue detection, significant correlations were found between these two methods with KSS. ROC analysis also showed PLR variations achieved high AUCs for driver fatigue detection, with high sensitivities and specificities, a comparative performance with HRV variations. However, a combination of PLR and HRV variations did not significantly increase the performance, suggesting that PLR variations, especially ΔMCV, could be potential indicators of driving fatigue in the future.

So far, no reliable commercialized driving fatigue detection system has been developed. The technology used in the present study lays a good foundation for the later development of such a detection system. It is expected that the system will be reliable, portable, sensitive and convenient to control, and can be applied to a variety of scenarios.

This study also has several limitations. As a preliminary study to determine the effectiveness of automated pupillometry to detect driving fatigue, a uniform sample who were all medical postgraduates with a fixed age, and a small sample size recruited from a single institution, are potential limitations. Then, limited degree of fatigue existed in the present fatigue model, the results in a more fatigued state remain unknown. Thirdly, an intervention outcome is not compared to a suitable control group that undergoes a different experience to assess effects of passage of time. Last, considering individual differences in pupil measures, thresholds for pupillary variables should be further validated through a large-scale, randomized, controlled trial in the future.

Pupillary light reflex variation may be a potential indicator in the detection of driving fatigue, achieving a comparative performance compared with the combination with heart rate variability. Further work may be involved in developing a commercialized driving fatigue detection system based on pupillary parameters.

## Data Availability Statement

The raw data supporting the conclusions of this article will be made available by the authors, without undue reservation.

## Ethics Statement

Approval for the study was obtained from the Ethics Committee of Second Affiliated Hospital of Zhejiang University School of Medicine (approval number: 2020-893). The patients/participants provided their written informed consent to participate in this study.

## Author Contributions

MZ and ZL designed the study. LS, LZ, DJ, and QZ performed the experiment. LS carried out statistical analyses and drafted the manuscript. MZ and LZ revised the manuscript for important intellectual content. All authors interpreted the study results and contributed with manuscript revisions.

## Funding

This research was supported by the Zhejiang Provincial Key Research and Development Program of China (2021C03073).

## Conflict of Interest

The authors declare that the research was conducted in the absence of any commercial or financial relationships that could be construed as a potential conflict of interest.

## Publisher's Note

All claims expressed in this article are solely those of the authors and do not necessarily represent those of their affiliated organizations, or those of the publisher, the editors and the reviewers. Any product that may be evaluated in this article, or claim that may be made by its manufacturer, is not guaranteed or endorsed by the publisher.

## Abbreviations

ACV: average constriction velocity
ADV: average dilation velocity
AUC: area under curve
HF: high frequency
HFnu: normalization of HF power by the formula: HF / (LF + HF) ^*^ 100
HRV: heart rate variability
LF: low frequency
LFnu: normalization of LF power by the formula: LF / (LF + HF) ^*^ 100
Lat: latency
Max: maximum pupil size
MCV: maximum constriction velocity
Min: minimum pupil size
%PLR: constriction percentage
SDNN: standard deviation of the NN intervals
T75: time to 75% recovery
Δ: differences in all variables from baseline to the measurement at each timepoint.
